# Ability of Different Bacteria from Grapevine to Colonize *Arabidopsis thaliana* Plants

**DOI:** 10.3390/plants15081151

**Published:** 2026-04-09

**Authors:** Olga A. Aleynova, Alexey A. Ananev, Nikolay N. Nityagovsky, Andrey R. Suprun, Alina A. Beresh, Alexandra S. Dubrovina, Konstantin V. Kiselev

**Affiliations:** 1 Laboratory of Biotechnology, Federal Scientific Center of the East Asia Terrestrial Biodiversity of Far Eastern Branch of the Russian Academy of Sciences, 690022 Vladivostok, Russia; aleynova@biosoil.ru (O.A.A.); ananev.all@yandex.ru (A.A.A.); niknit1996@gmail.com (N.N.N.); suprun.hi@gmail.com (A.R.S.); a.beresh@mail.ru (A.A.B.); dubrovina@biosoil.ru (A.S.D.); 2 Advanced Engineering School, Far Eastern Federal University, 690922 Vladivostok, Russia; 3 Institute of the World Ocean, Far Eastern Federal University, 690922 Vladivostok, Russia

**Keywords:** bacterial communities, Endophytes, metagenomic analysis, plant–endophyte interactions, plant colonization

## Abstract

This study investigates the impact of inoculating seeds with bacterial endophytes isolated from *Vitis amurensis* Rupr. on endophytic community composition in *Arabidopsis thaliana* (L.) Heynh. Ten bacterial isolates of the genera *Agrobacterium*, *Bacillus*, *Curtobacterium*, *Erwinia*, *Frondihabitans*, *Gordonia*, *Pantoea*, *Pseudomonas*, *Sphingomonas*, and *Xanthomonas* were applied to seeds and some visible phenotypic effects were observed on plant growth after two weeks. High-throughput sequencing of 16S rRNA revealed that the native endophytic microbiome of *A. thaliana* was dominated by Gammaproteobacteria, Actinomycetes, Bacteroidia, and Alphaproteobacteria. The key families were *Microscillaceae, Chitinophagaceae*, *Rhizobiaceae*, *Rhodanobacteraceae*, *Nocardioi-daceae*, *Nocardiaceae*, *Xanthomonadaceae, Devosiaceae*, *Microbacteriaceae*, *Crocinitomi-caceae*, *Pseudomonadaceae*, *Solimonadaceae*, *Comamonadaceae*, *Caulobacteraceae*, and *Micrococcaceae*. *Arabidopsis* seed inoculation with *Agrobacterium* sp. R8SCh-B12, *Curtobacterium* sp. P7SA-B3, and *Gordonia aichiensis* P6PL2 significantly reduced alpha diversity (Shannon index) and altered beta diversity relative to controls, indicating strong community restructuring. These three isolates, along with *Pseudomonas* sp. R8SCh-B2, *Sphingomonas* sp. RA62c-B5, *Xanthomonas* sp. R7SCh-B6, and *Bacillus velezensis* AMR25, successfully colonized the plant tissues, as evidenced by significant increases in genus-specific amplicon sequence variants, ASVs (up to 17,820-fold for *Curtobacterium* sp. ASV33). In contrast, *Pantoea* sp. P7SCH-B5, *Erwinia* sp. R8SCh-B3, and *Frondihabitans* sp. RA62c-B2 failed to colonize *A. thaliana*, despite being applied to the seeds, suggesting the existence of mechanisms restraining colonization. These findings demonstrate that only a subset of grapevine-derived endophytes can effectively colonize *A. thaliana*, and that successful colonization correlates with significant shifts in the native microbiome, even in the absence of overt phenotypic changes. This emphasizes the importance of strain-specific compatibility in plant–endophyte interactions. Thus, we report the first descriptions of several novel endophytes that colonized *Arabidopsis* plants and establish a convenient model to investigate plant–bacterial interactions.

## 1. Introduction

Endophytes are microorganisms that live inside their host plant without causing any visible symptoms during their entire life cycle [1]. The colonization of plant tissues by endophytes represents a critical interface in plant–microbe symbiosis, influencing host physiology, stress resilience, and developmental trajectories [2,3]. The role of bacterial endophytes in promoting plant growth has been extensively documented in crops such as maize, rice, and tomato, etc. [4].

Previous studies have demonstrated that endophytes isolated from wild-growing grapevine *Vitis amurensis* Rupr.—a species renowned for its extreme abiotic stress tolerance, harbor a diverse assemblage of bacteria with potential bio-stimulatory and biocontrol properties [5]. These bacteria from different genera as *Agrobacterium*, *Bacillus*, *Pseudomonas*, *Sphingomonas*, and *Curtobacterium*, were originally isolated from surface-sterilized grapevine leaves and stems, confirming their internal, endophytic lifestyle [5]. However, their ability to successfully colonize distantly related plant hosts such as *Arabidopsis thaliana* (L.) Heynh., a model organism with a well-characterized genome and well-defined endophytic microbiome [6], has not been systematically evaluated. *A. thaliana* is useful for molecular studies of the mechanisms involved in the interaction between plants and endophytes. However, in order to create a scientific basis for studying the effects of endophytes on plants, it is necessary to find endophytes that can colonize *Arabidopsis*.

Numerous studies have documented bacterial interactions with *Arabidopsis* plants, although the majority focus on the effects of pathogenic microorganisms on this model species (Nishimura et al., 2010) [7]. There are only a few studies that have examined the possibility of colonization by bacteria and fungi in *Arabidopsis* plants. For example, the bacterium *Gluconacetobacter diazotrophicus* had a positive effect on the growth of *Arabidopsis* plants during endophytic colonization by enhancing whole plant photosynthesis and improving instantaneous whole canopy water-use efficiency [8]. Inoculation of *Arabidopsis* seeds with *Streptomyces* strain coa1 spores resulted in both intercellular and intracellular root colonization, leading to alterations in root hormonal balance and antibiotic synthesis (van der Meij et al., 2018) [9]. Colonization by another bacterium, *Bacillus subtilis*, yielded similar results: root colonization was observed, accompanied by the activation of multiple biosynthetic gene clusters involved in antibiotic production (Maan et al., 2021) [10].

Also, *A. thaliana* was used as a model for more detailed dissection of the dark septate fungal endophyte symbiosis [11]. In Kim et al., 2017 [12], it was shown that colonization by endophyte fungus *Piriformospora indica* leads to early flowering in *A. thaliana*, likely by triggering gibberellin biosynthesis.

Moreover, some studies have shown that external environmental conditions (such as UV-A radiation) can affect the effectiveness of fungal endophyte plant colonization [13], which is also an area that is still poorly understood. However, this knowledge can increase the effectiveness of using endophytes in agriculture. Therefore, the development of model systems for studying endophytes is highly relevant at the current stage of advancing knowledge regarding plant–bacterium interactions.

In this study, we inoculated *A. thaliana* seeds with ten bacterial endophytes belonging to different genera isolated from *V. amurensis*, because the effects of bacterial endophytes on this model species are poorly understood. We employed metagenomic high-throughput 16S rRNA and *ITS1* amplicon sequencing to assess bacterial endophytes impact on both bacterial and fungal endophytic communities in *A. thaliana*. This approach is limited by the challenge of designing suitable primers capable of amplifying the target DNA regions of the studied organisms. Therefore, careful selection and validation of primers is essential.

Our metagenomic results reveal significant differences in colonization efficiency in different grapevine bacterial strains. Some strains—such as, *Agrobacterium* sp. R8SCh-B12, *Curtobacterium* sp. P7SA-B3, and *Sphingomonas* sp. RA62c-B5—robustly colonized *A. thaliana* tissues and dominated the endophytic bacterial community. In contrast, other strains such as *Pantoea* sp. P7SCH-B5, *Erwinia* sp. R8SCh-B3, and *Frondihabitans* sp. RA62c-B2 had no noticeable presence in the treated plants. Thus, based on metagenomic data and real-time PCR, we report the first description of several novel endophytes (e.g., from genera *Curtobacterium*, *Frondihabitans*, *Gordonia*, or *Xanthomonas*) colonized *Arabidopsis* plants. Moreover, the relative abundance of DNA from these bacterial species suggests their differing capacities to colonize *Arabidopsis*. Thus, this work establishes a convenient experimental model for the detailed study of plant–bacteria interactions, enabling further mechanistic insights.

## 2. Results and Discussion

### 2.1. Effect of Inoculating Seeds with Bacterial Endophytes on A. thaliana Growth and Development

Ten bacterial strains representing distinct genera from the endophyte collection of the Biotechnology laboratory were selected for the experiments (Table 1). These endophytes were previously isolated from wild-growing grapevine *V. amurensis* and included the following genera: *Agrobacterium* (or *Rhizobium*), *Bacillus*, *Curtobacterium*, *Erwinia*, *Frondihabitans*, *Gordonia*, *Pantoea*, *Pseudomonas*, *Sphingomonas*, *Xanthomonas*. It is important to note that initially all the strains in the 16S rRNA gene sequence belonged to the corresponding genera [5], then full-scale sequencing was performed for some of them and the species were determined more precisely to the species (Table 1). Thus, a wide range of pathogenic bacteria and bacteria capable of stimulating plant growth were used in this paper.

After seven days of growth on the Murashige and Skoog (MS) medium, the seedlings were transferred to the soil-filled pots and cultivated for two weeks. Subsequently, DNA was isolated from the terrestrial parts of plants and endophyte community composition were analyzed.

**Figure 1:**
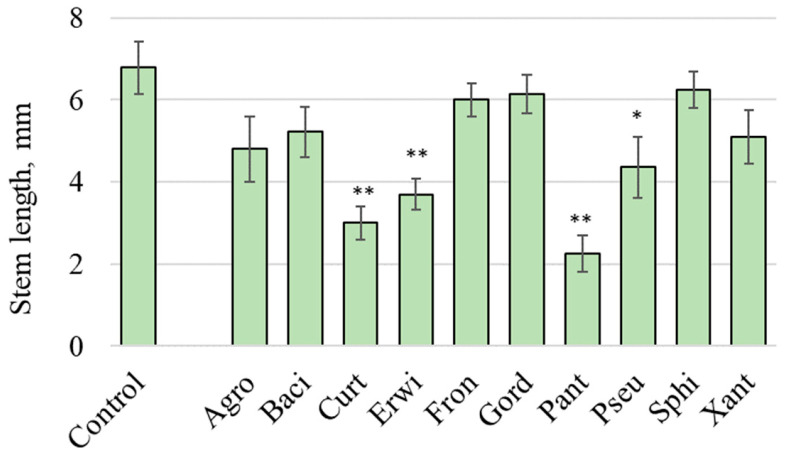
The stem length of 7-day-old *Arabidopsis thaliana* seedlings were measured after being grown on a ½ Murashige and Skoog (MS) nutrient medium with endophytic bacteria from wild grapevine *Vitis amurensis* distributed on the surface of the MS medium. Control—uninoculated *A. thaliana* seedlings. Agro—treatment by *Agrobacterium* sp.; Baci—*Bacillus velezensis*; Curt—*Curtobacterium* sp.; Erwi—*Erwinia* sp.; Fron—*Frondihabitans* sp.; Gord—*Gordonia aichiensis*; Pant—*Pantoea* sp.; Pseu—*Pseudomonas* sp.; Sphi—*Sphingomonas* sp.; Xant—*Xanthomonas* sp. * *p* < 0.05; ** *p* < 0.01 versus values of stem length in the Control (Student’s *t* test).

During plant cultivation, no external disease symptoms (e.g., chlorosis, necrosis, or leaf spots) were observed. However, certain endophytic bacteria, including *Curtobacterium* sp., *Erwinia* sp., *Pantoea* sp., and *Pseudomonas* sp., significantly inhibited *A. thaliana* stem length, as shown in Figure 1. The observed significant reduction in *A. thaliana* stem length (Figure 1) may be attributed to the pathogenic nature of the bacterial isolates used; however, further research is required for confirmation. This hypothesis is supported by the existing literature indicating that members of these bacterial genera are recognized plant pathogens (Table 1). Additionally, long-term experiments [14] have demonstrated that exposure to these isolates resulted in the death of *Arabidopsis* plants, which failed to survive beyond two months.

**Table 1:** The used bacterial endophytes from wild growing grapevine *Vitis amurensis*.

Endophyte Name, Genebank	Known Properties of Used Endophytes or Other Representatives of Same Genus
*Agrobacterium* sp., R8SCh-B12, MZ424738	Well known for its ability to transfer DNA between itself and plants causes crown-gall disease. The disease is characterized by a tumor-like growth or gall on the roots and shoots. Therefore, it is customary to classify them as plant pathogens [15].
*Bacillus velezensis*, AMR25, CP140115.1, [16]	Producer of antimicrobial metabolites. Demonstrated efficacy in inhibiting both phytopathogenic fungi and bacteria [16,17].
*Curtobacterium* sp., P7SA-B3, MZ424740	A cosmopolitan terrestrial taxon, with isolates derived primarily from plant and soil habitat. Significant pathogenic bacteria of soybeans and other agricultural crops [18].
*Erwinia* sp., R8SCh-B3, MZ424741	Mostly plant pathogenic species. Many infect woody plants [19].
*Frondihabitans* sp., RA62c-B2, JX949771	Non-spore-forming and non-motil genus of bacteria, isolated from the leaf litters, thallus of the lichen, sap of the maple tree, rhizosphere soil of the plant coastal hog fennel [20].
*Gordonia aichiensis*, P6PL2, PRJNA1267753, [21]	This genus is widely distributed and can be found in soil, water, plants, and medical specimens. *G. aichiensis* stimulates plant growth (rice) [21].
*Pantoea* sp., P7SCH-B5, PZ204378	This genus includes at least 20 species. Is wildly found in various natural environments, such as plants, water, and soil and is generally considered as a plant pathogen [22].
*Pseudomonas* sp., R8SCh-B2, MZ424743	Demonstrate a great deal of metabolic diversity and consequently are able to colonize a wide range of niches and hosts. The best studied species include *P. aeruginosa* in its role as an opportunistic human pathogen, the plant pathogen *P. syringae*, the soil bacterium *P. putida*, the plant growth-promoting *P. fluorescens*, *P. lini*, *P. migulae*, and *P. graminis* [23].
*Sphingomonas* sp., RA62c-B5, PX909750	Isolated from many different land, water habitats, as well as from plant root systems, clinical specimens, etc., e.g., *Sphingomonas melonis* can be naturally enriched in certain rice cultivars, confers diseases resistance against a bacterial pathogen and is vertically transmitted among plant generations via their seeds [24].
*Xanthomonas* sp., R7SCh-B6, MZ424744	There are at least 27 plant associated *Xanthomonas* spp., that all together infect at least 400 plant species. Different species typically have specific host and/or tissue range and colonization strategies [25].

### 2.2. The Effect of Arabidopsis Seeds Inoculating with Grapevine Bacterial Endophytes on the Composition of A. thaliana Bacterial Endophytes

For DNA extraction, *Arabidopsis* plants were surface-sterilized via sequential washing: first with 10% hydrogen peroxide, then with 70% ethanol, and finally rinsed five times with sterile distilled water. The efficacy of sterilization was confirmed by plating the final wash water on Reasoner’s agar 2A (R2A) and potato dextrose agar (PDA) media, with no bacterial or fungal growth detected. Therefore, we propose that the signals detected in the isolated DNA originate from *Arabidopsis* endophytes.

**Figure 2:**
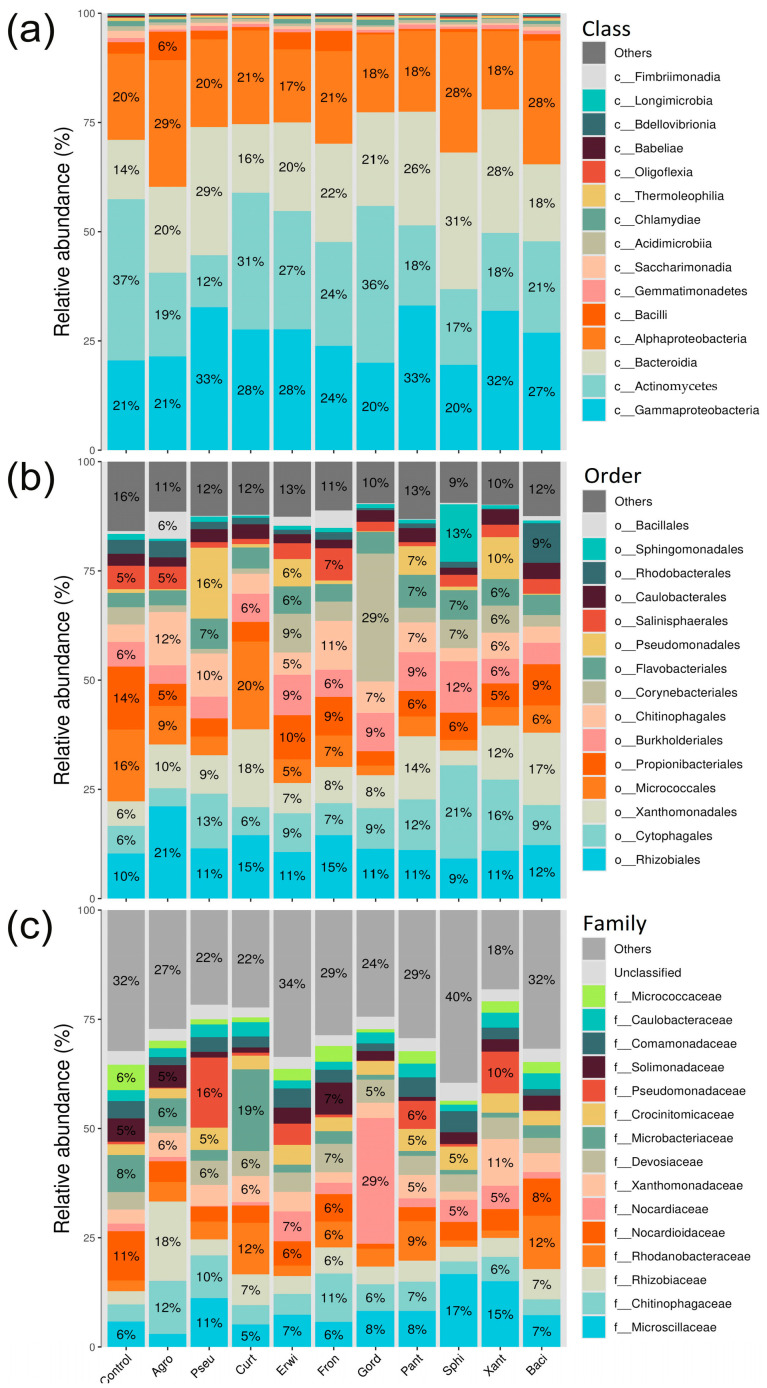
Composition of endophytic bacterial communities in *Arabidopsis thaliana* at class (**a**), order (**b**), and family (**c**) levels after endophytes inoculation. The top 15 classes, orders, or families are shown. The remaining taxa are grouped into the other category. Agro—treatment by *Agrobacterium* sp.; Pseu—*Pseudomonas* sp.; Curt—*Curtobacterium* sp.; Erwi—*Erwinia* sp.; Fron—*Frondihabitans* sp.; Gord—*Gordonia aichiensis*; Pant—*Pantoea* sp.; Sphi—*Sphingomonas* sp.; Xant—*Xanthomonas* sp.; Baci—*Bacillus velezensis*.

As a result of high-performance sequencing, after purification from low-quality reads, from 51,715 to 63,061 filtered reads per sample were obtained, which indicates high support for the obtained sequencing data (Table S1). Next, taxonomic identification was conducted using the QIIME 2 BLAST+ algorithm, leveraging the SILVA database for 16S sequences and the UNITE database for ITS sequences. The dominant bacterial classes in *A. thaliana* belonged to Gammaproteobacteria, Actinomycetes, Bacteroidia, and Alphaproteobacteria (Figure 2a). Each of the dominant taxonomic classes across all samples accounted for more than 12% of the total reads (ranging from 12% to 37%), and collectively, these classes represented over 85% of all analyzed sequencing reads (Figure 2a).

The higher number of orders and families complicates the identification of dominant groups. Regarding orders, the most abundant groups included Rhizobiales, Cytophagales, Xanthomonadales, Micrococcales, and Propionibacteriales (Figure 2b). These taxonomic orders are present in all samples, and their total proportion of reads accounts for 53% in untreated control *Arabidopsis* plants (Figure 2b).

Identifying dominant classes proves even more challenging, as the proportion of reads attributed to individual families does not exceed 19%, with the sole exception of the *Nocardioidaceae* family (29%). However, this elevated proportion is observed exclusively in a sample containing *Gordonia aichiensis*, a bacterium belonging to this family. Therefore, we identified 15 bacterial families as the predominant taxa: *Microscillaceae*, *Chitinophagaceae*, *Rhizobiaceae*, *Rhodanobacteraceae*, *Nocardioidaceae*, *Nocardiaceae*, *Xanthomonadaceae*, *Devosiaceae*, *Microbacteriaceae*, *Crocinitomicaceae*, *Pseudomonadaceae*, *Solimonadaceae*, *Comamonadaceae*, *Caulobacteraceae*, and *Micrococcaceae*. The proportion of reads from these families ranged from 55% to 81% of the total reads obtained (Figure 2c).

At the class and order levels, differences are only weakly discernible, whereas they become more pronounced at the family level. For instance, treatment with *Agrobacterium* sp. resulted in a marked increase in the abundance of the *Rhizobiaceae* family, which includes this species. Also, an increase in the abundance of the *Nocardioidaceae* family was detected after treatment with *G. aichiensis*. *G. aichiensis* is a species classified within this bacterial family (Figure 2c).

**Figure 3:**
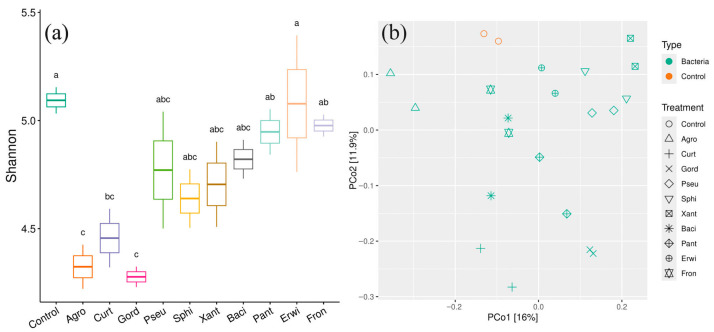
Shannon indexes of endophytic bacterial communities in *Arabidopsis thaliana* plants (**a**); PCoA based on Bray–Curtis dissimilarity for endophytic bacterial after bacterial treatment (**b**). Different lowercase letters indicate significant differences among Shannon indexes. Ellipses in PCoA plots show the 95% confidence intervals of a multivariate *t*-distribution. Agro—treatment by *Agrobacterium* sp.; Curt—*Curtobacterium* sp.; Gord—*Gordonia aichiensis*; Pseu—*Pseudomonas* sp.; Sphi—*Sphingomonas* sp.; Xant—*Xanthomonas* sp.; Baci—*Bacillus velezensis*; Pant—*Pantoea* sp.; Erwi—*Erwinia* sp.; Fron—*Frondihabitans* sp.

It is important to note that the inoculation with *Pseudomonas* sp., *Sphingomonas* sp., *Xanthomonas* sp., *B. velezensis* (*Bacillus*), *Pantoea* sp., *Erwinia* sp., and *Frondihabitans* sp. bacteria did not significantly alter the diversity of endophytic bacterial communities, as assessed by the Shannon alpha-diversity index (Figure 3a). In contrast, inoculation with *Agrobacterium* sp., *Curtobacterium* sp., and *G. aichiensis* significantly decreased endophytic bacterial diversity (Figure 3a). PCoA based on Bray–Curtis dissimilarity revealed a distinct clustering of bacterial com-munities by treatment, supported by PERMANOVA (R2 = 0.73, *p* < 0.001) (Figure 3b). Also, beta diversity confirms the results obtained on alpha diversity that plants treated with *Agrobacterium* sp., *Curtobacterium* sp., and *G. aichiensis* differed the most from the control plants (Figure 3b).

**Figure 4:**
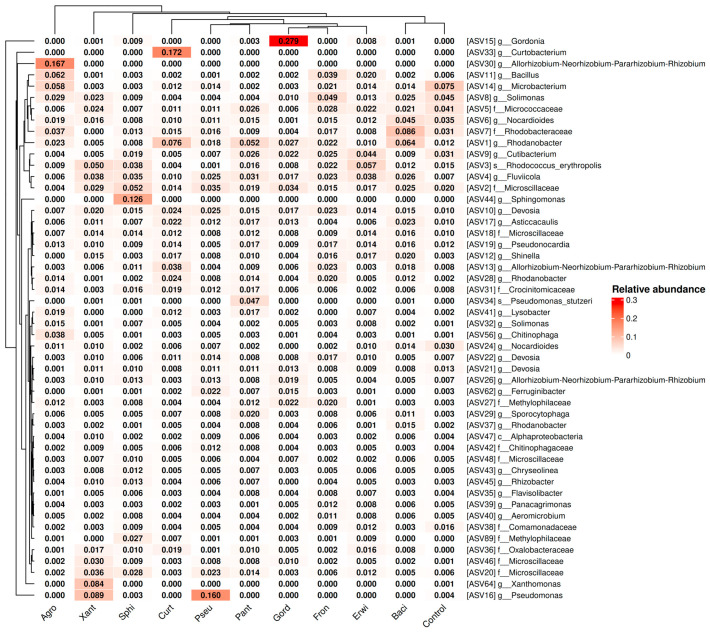
Heat maps illustrating the relative abundance of endophytic bacteria at the genus level were constructed, focusing on the significant taxa identified through next-generation sequencing (NGS) in *Arabidopsis thaliana* plants after endophytic bacteria inoculation. The top 50 amplicon sequence variant (ASVs) with the highest abundance are shown. The sequences of ASVs are presented in the Supplementary Table S2. Agro—treatment by *Agrobacterium* sp.; Xant—*Xanthomonas* sp.; Sphi—*Sphingomonas* sp.; Curt—*Curtobacterium* sp.; Pseu—*Pseudomonas* sp.; Pant—*Pantoea* sp.; Gord—*Gordonia aichiensis*; Fron—*Frondihabitans* sp.; Erwi—*Erwinia* sp.; Baci—*Bacillus velezensis*.

Next, we conducted a detailed analysis of the bacterial genus composition based on amplicon sequence variants, ASVs (Figure 4). It is important to note that a single genus may be represented by multiple ASVs, which typically differ by few nucleotide substitutions. The ASV sequences are provided in Supplementary Table S2. The ASV analysis shows in more detail that the addition of *Agrobacterium* sp., *Curtobacterium* sp., *G. aichiensis* (*Gordonia*), *Pseudomonas* sp., *Sphingomonas* sp., *Xanthomonas* sp., and *B. velezensis* (*Bacillus*) increased ASV corresponding to their genus: *Agrobacterium* sp.—ASV30, *Curtobacterium* sp.—ASV33, *G. aichiensis* (*Gordonia*)—ASV15, *Pseudomonas* sp.—ASV16, *Sphingomonas* sp.—ASV44, *Xanthomonas* sp.—ASV64, and *B. velezensis* (*Bacillus*)—ASV297 (Figure 4).

**Figure 5:**
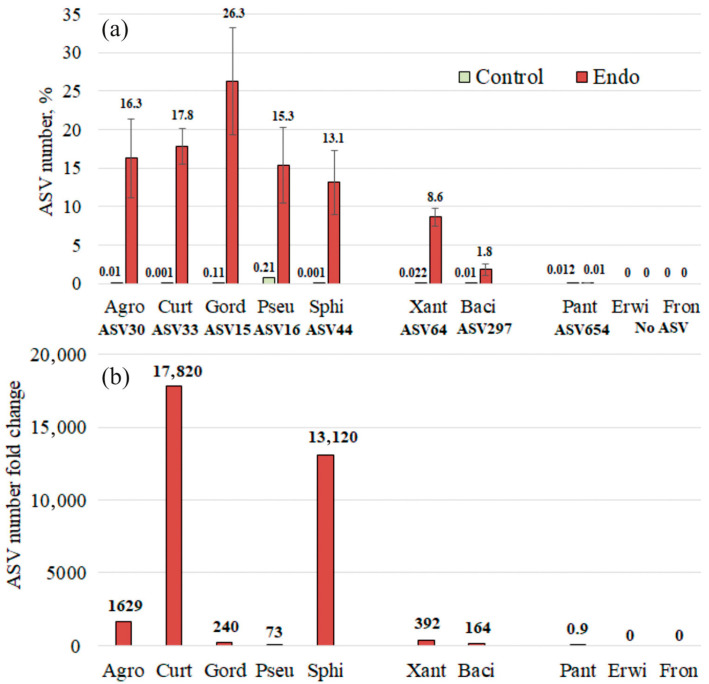
Changes in the amplicon sequence variant (ASV) number (**a**) and ASV number fold change (**b**) after bacterial endophytes treatment (+Endo). Agro—treatment by *Agrobacterium* sp.; Curt—*Curtobacterium* sp.; Gord—*Gordonia* sp.; Pseu—*Pseudomonas* sp.; Sphi—*Sphingomonas* sp.; Xant—*Xanthomonas* sp.; Baci—*Bacillus* sp.; Pant—*Pantoea* sp.; Erwi—*Erwinia* sp.; Fron—*Frondihabitans* sp.

Normally, *Pantoea* sp. bacteria (ASV654) are present in plants (Table S2). However, inoculation of *A. thaliana* seeds with this bacterium did not result in an increase in *ASV654* abundance in two-week-old plants (Table S2; Figure 5). This suggests that *Pantoea* sp. either failed to colonize *Arabidopsis* or was unable to compete with the resident microbial community.

At the same time, ASV of *Erwinia* sp. and *Frondihabitans* sp. bacteria were not present in the analyzed samples at all. Therefore, we can conclude with greater certainty that these bacteria do not penetrate *Arabidopsis* plants. If they were capable of colonization, they would be detected under normal conditions; however, no evidence of their presence was observed (Table S2).

Next, we presented the data on ASV, the corresponding bacteria that were used in the experiments, separately in Figure 5a. Also, we calculated the fold-increase for each ASV by dividing the relative abundance presented in Figure 5a (percentage) of each ASV in the plant sample inoculated with endophytes by its corresponding value in the control (untreated) sample (Figure 5b).

Here it is shown more clearly that addition of *Agrobacterium* sp., *Curtobacterium* sp., *G. aichiensis* (*Gordonia*), *Pseudomonas* sp., *Sphingomonas* sp., *Xanthomonas* sp., and *B. velezensis* (*Bacillus*) increased ASV corresponding to their genus: *Agrobacterium* sp.—1629 times, *Curtobacterium* sp.—17820 times, *G. aichiensis* (*Gordonia*)—240 times, *Pseudomonas* sp.—73 times, *Sphingomonas* sp.—13 120 times, *Xanthomonas* sp.—392 times, and *B. velezensis* (*Bacillus*)—164 times (Figure 5b). While the addition of *Pantoea* sp., *Erwinia* sp., and *Frondihabitans* sp. did not lead to an increase in the corresponding ASV in the plant samples (Figure 5a,b).

The absence or low abundance of ASVs corresponding to *Pantoea* sp., *Erwinia* sp., and *Frondihabitans* sp. may be attributed to primer mismatch, which prevented their efficient amplification. However, as shown in Figure S1, the primers were fully compatible with the 16S rRNA gene sequences of these taxa and amplification of the 16S rRNA gene from the DNA of these endophytes, using the same primers employed in the metagenomic sequencing, was successful. This suggests that these bacterial genera were present in the samples at very low abundances.

To validate the metagenomic findings presented in Figure 5b, we performed real-time PCR to assess the presence and abundance of bacterial DNA in control (untreated) plants and in plants following treatment with the all respective bacterial endophytes. The data confirmed that inoculation with *Agrobacterium* sp., *Sphingomonas* sp., and *Sphingomonas* sp. led to a dramatic and highly significant increase in 16S rRNA gene amplification specific to these bacterial strains (Figure S2). Bacterial treatment by *Gordonia* sp., *Pseudomonas* sp., *Xanthomonas* sp., and *Bacillus* sp. also significantly enhances the 16S rRNA gene amplification specific to these bacterial strains, but to a lesser degree (Figure S2). In contrast, the amplification signal for *Pantoea* sp., *Erwinia* sp., and *Frondihabitans* sp. did not differ significantly from that detected in control plants (Figure S2).

### 2.3. The Effect of Arabidopsis Seeds Inoculating with Grapevine Bacterial Endophytes on the Composition of A. thaliana Fungal Endophytes

It is known that plants are also inhabited by microscopic fungi, so it was important to understand how the invasion of bacteria affects the normal distribution of fungi in *Arabidopsis* plants. To do this, we performed metagenomic analysis of the *ITS1* regione PCR products. As a result of high-performance sequencing, after purification from low-quality reads, from 39,777 to 59,295 filtered reads per sample were obtained, which indicates high support for the obtained sequencing data (Table S1).

**Figure 6:**
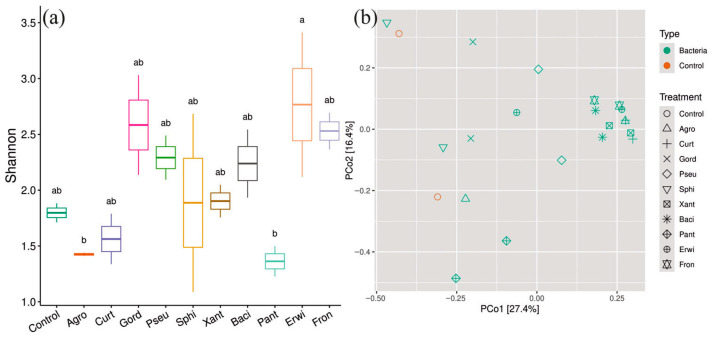
Shannon indexes of endophytic fungal communities in *Arabidopsis thaliana plants* (**a**); PCoA based on Bray–Curtis dissimilarity for endophytic fungi after bacterial inoculation (**b**). Different lowercase letters indicate significant differences among Shannon indexes. Ellipses in PCoA plots show the 95% confidence intervals of a multivariate *t*-distribution. Agro—treatment by *Agrobacterium* sp.; Curt—*Curtobacterium* sp.; Gord—*Gordonia aichiensis*; Pseu—*Pseudomonas* sp.; Sphi—*Sphingomonas* sp.; Xant—*Xanthomonas* sp.; Baci—*Bacillus velezensis*; Pant—*Pantoea* sp.; Erwi—*Erwinia* sp.; Fron—*Frondihabitans* sp.

It was shown that the addition of all bacterial endophytes had no significant impact on the alpha diversity of the indigenous fungal community (Figure 6a). However, fungal community structures exhibited significant shift across treatments, as evidenced by PCoA and PERMANOVA analysis (R2 = 0.62, *p* < 0.01) (Figure 6b).

**Figure 7:**
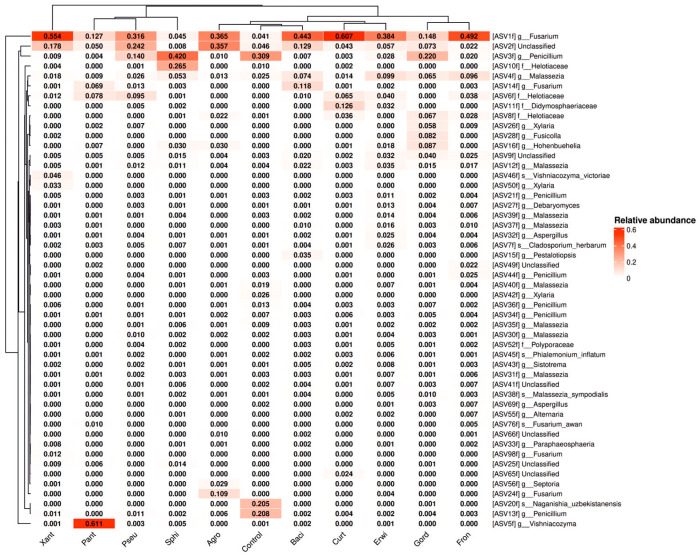
Heat maps illustrating the relative abundance of endophytic fungi at the genus level were constructed, focusing on the significant taxa identified through next-generation sequencing (NGS) in *A. thaliana* plants after endophytic bacteria inoculation. The top 50 ASVs with the highest abundance are shown. The sequence of ASVs are presented in the Supplementary Table S2. Agro—treatment by *Agrobacterium* sp.; Curt—*Curtobacterium* sp.; Gord—*Gordonia aichiensis*; Pseu—*Pseudomonas* sp.; Sphi—*Sphingomonas* sp.; Xant—*Xanthomonas* sp.; Baci—*Bacillus velezensis*; Pant—*Pantoea* sp.; Erwi—*Erwinia* sp.; Fron—*Frondihabitans* sp.

When inoculated with all endophytic bacteria in *Arabidopsis* plants, the representation ASV3f and ASV13f of the genus *Penicillium* and ASV20f of the species *Naganishia uzbekistanensis* decreases (Figure 7). At the same time, inoculation with used endophytes resulted in a significant increase in *Fusarium* ASV (1f, 14f, 24f) levels in samples, with fold changes ranging from 4- to 15-fold for ASV1f (Figure 7). The sole exception was observed in the treatment with *Sphingomonas* sp., where no increase in *Fusarium* ASV and no decrease in ASV of the genus *Penicillium* was detected (Figure 7).

While several studies have demonstrated that certain *Penicillium* species can stimulate plant growth [26], there is currently no available data on the effects of *Naganishia uzbekistanensis* on plants. It is plausible that these fungi, as part of the normal healthy microbiological flora, act as antagonists to other pathogenic microorganisms; however, this hypothesis requires further experimental validation.

Data indicating an increase in *Fusarium* ASVs abundance are of particular interest. *Fusarium* species constitute a genus of filamentous fungi recognized as economically important plant pathogens, responsible for a wide range of diseases that adversely affect crop health and reduce agricultural yields.

Therefore, it can be hypothesized that certain inoculated bacteria exhibit pathogenic properties, particularly *Curtobacterium* sp. This strain is associated with the highest *Fusarium* ASVs abundance (Figure 6) and the most pronounced inhibition of *Arabidopsis* seedling stem growth (Figure 1).

## 3. Conclusions

This study based on metagenomic and real-time PCR data illustrates it is possible to distinguish 3 groups of bacteria by their ability to colonize *Arabidopsis* plants. The first group: bacteria that quickly colonize plants (*Agrobacterium* sp., *Curtobacterium* sp., and *Sphingomonas* sp.). The second group: weakly colonize (*G. aichiensis*, *Pseudomonas* sp., *Xanthomonas* sp., and *B. velezensis* or *Bacillus*); the third group: bacteria that do not colonize at all (*Pantoea* sp., *Erwinia* sp., and *Frondihabitans* sp.).

It is important to note that the selected bacteria in the experiments were previously isolated from externally sterilized grapevine leaves and stems, and therefore are characteristic of the internal microbiota of grapevines. This may be the reason why *Pantoea* sp., *Erwinia* sp. and *Frondihabitans* sp. do not thrive on *Arabidopsis*: they may be specific endophytes of grapevines, although further research is needed to confirm this hypothesis.

Also, we described the conditions under which the high ability of some bacteria (*Agrobacterium* sp., *Curtobacterium* sp., and *Sphingomonas* sp.) to penetrate into the interior of *Arabidopsis* plants was shown. It is known that endophytes with detrimental effects on mono-association with the host colonize roots more aggressively than those with beneficial activities, and dominate in natural root samples [27]. In our case, two out of three well-colonizing endophytes are potentially pathogenic isolates [11,15], which partially confirms this observation.

Most of the bacteria used penetrated poorly or did not get into the *Arabidopsis* endophytic microflora at all. It is possible to increase the ability of bacteria to penetrate by using special environmental conditions or by adding certain chemicals (polyphenols, polysaccharides, etc.), but this is also important and requires additional research. Furthermore, using the described bacteria with different ability to colonize *Arabidopsis* plants, it is possible to study both bacterial and plant genes that are responsible for plant protection and interaction with microorganisms [28].

We conclude that our findings provide an initial framework for future research on endophyte–*Arabidopsis* interactions, using a well-characterized *Arabidopsis* plant together with several novel bacterial endophytes that were first demonstrated to colonize *Arabidopsis* plants (*Curtobacterium* sp., *Frondihabitans* sp., *Gordonia aichiensis*, and *Xanthomonas* sp.). This model offers substantial experimental utility for work on engineering endophytic consortia to enhance plant growth. It underscores the importance of selecting bacterial strains based on their host adaptation capacity rather than solely on origin or in vitro bioactivity.

## 4. Materials and Methods

### 4.1. Plant Material

For experiments, we used seeds of *A. thaliana* (ecotype Columbia-0), which were stored in our laboratory. To sterilize the seeds, we exposed them for 40–50 min to chlorine vapors by adding 3 mL of concentrated HCl to 100 mL of bleach (Sayanskhimplast, 7%, Sayansk, Russia).

The sterilized seeds were then germinated in Petri dishes with ^1^/_2_ MS medium [29] with a pH of 5.6, solidified with 0.8% agar, for germination and placed in an environmental chamber at a temperature of 22 °C and a light intensity of 120 μmol m^−2^ s^−1^. After 7 days of growth on the MS medium, the seedlings were transferred to the pots with commercially available soil (“Universalny”, Fasko, Zelenograd, Russia). The soil consisted of riding peat, lowland peat, sand, limestone (dolomite) flour, and complex mineral fertilizer with microelements. The content of nutrients available to plants (mg/kg) was not less than: nitrogen—350; phosphorous—400; potassium—500; pH: 6–7 (https://fasko.ru/product-category/grunt/grunt_universalnyy, accessed on 1 April 2026). Pots with seedlings were placed in an environmental control chamber (BPC500H, Fujian Jiupo Biotechnology Co., Ltd., Fujian Province, Fuzhou, China). The chamber maintained a 16/8 h day/night cycle, with a temperature of 22 °C and a light intensity of 120 μmol m^−2^ s^−1^.

### 4.2. Bacterial Inoculation

Endophytic bacteria were obtained from the surface-sterilized leaves and stems of wild-growing *Vitis amurensis* grapevines growing in the unprotected areas of the Primorsky Territory, Russia. The process of isolating individual endophytic strains has been thoroughly documented in a previous publication [5,30]. Briefly, the leaves and stems of *V. amurensis* were briefly washed under running water, soaked in 70% ethanol for one minute and 10% H_2_O_2_ for two minutes, and then washed five times in sterile distilled water. Next, a 150 mg fragment of sterile tissue was homogenized in a laboratory mortar with diameter of 10 cm and diluted with 200 μL of sterile water. Then, 70 μL of the resulting solution was plated on R2A medium and incubated at 25 °C for 24 h.

The DNA of various bacterial colonies was isolated using a modified version of the CTAB-spin method [31]. The bacterial 16S rRNA gene was amplified using universal bacterial primers (8F, 5′AGAGTTTGATCMTGGCTCAG and 1522R, 5′AAGGAGGTGATCCARCCGCA) to generate approximately 1500 bp PCR products [32]. The PCR products were then sequenced on an ABI 3130 Genetic Analyser (Applied Biosystems, Foster City, CA, USA) and analyzed using the BLAST program. Sequence analysis was performed by multiple sequence alignment using the Clustal X program [33], with a sequence identity of ≥99% used as the threshold for taxonomic identification.

We used the most prevalent endophytic species in wild grapes *V. amurensis*—these are bacteria *Rhizobium* (*Agrobacterium*) sp., *Bacillus* sp., *Curtobacterium* sp., *Erwinia* sp., *Frondihabitans* sp., *Gordonia* sp., *Pantoae* sp., *Pseudomonas* sp., *Sphingomonas* sp., *Xantomonas* sp. The bacterial isolates were placed in 20 mL of liquid nutrient medium R2A medium (PanReac, AppliChem, Darmstadt, Germany) and incubated for 24–48 h at 28 °C 150 rpm on an orbital shaker BioSan ES 20/60 (Riga, Latvia) raising the final concentration of 10^7^ UFC/mL. Next, 100 µL of each bacterial isolate was surface distributed by spreading with a sterilized loop on Petri dishes (diameter 12 cm) with ^1^/_2_ MS medium.

Under aseptic conditions, *Arabidopsis* seeds sterilized with chlorine vapors were sown on Petri dishes inoculated with endophytes. After 7–8 days of plant endophytic interactions in Petri dishes, the seedlings were transferred to pots filled with commercially available rich soil (“Universalniy”) in an environmental control chamber (BPC500H) kept on a 16/8 h light/darkness cycle at 22 °C and a light intensity of 120 μmol m^−2^s^−1^. After 14 days of growth in pots with soil, DNA was isolated from *Arabidopsis* plants that had previously been grown using grape endophytic bacteria for metagenomic analysis.

### 4.3. DNA Extraction, 16S/ITS Metagenomic Sequencing, and Quantative Real-Time PCR (qPCR)

DNA for NGS was extracted from two weeks old *A. thaliana* rosettes using the CTAB-spin technique as described previously [31]. Plants were surface-sterilized prior to DNA extraction by sequential washing: first in 10% hydrogen peroxide for 2 min, followed by 70% ethanol for 3 min, and finally rinsed five times with sterile distilled water. Sterilization efficacy was confirmed by plating the final wash water on R2A and PDA media; no bacterial or fungal growth was detected. Two experiments were conducted to evaluate the effect of endophyte treatment. For each treatment and the control group, DNA was isolated from a single plant exhibiting no visible disease symptoms (two biological repeats).

For advanced sequencing using the Illumina technology, the DNA samples were sent to Syntol in Moscow, Russia. Sequenced bacterial and fungal libraries were constructed according to a detailed protocol provided by the manufacturer of the sequencing platform “16S Metagenomic Sequencing Library Preparation” (Part # 15,044,223 Rev. B; Illumina, San Diego, CA, USA). This protocol can also be used for the preparation of a fungal library. Bacterial *16S* rRNA regions and fungal *ITS1* rDNA regions were amplified from the samples using the primers 515F, 5′GGTAATACGKAGGKKGCDAGC and 806R 5′RTGGACTACCAGGGTATCTAA described earlier [31]. Amplicons were indexed using the Nextera^®^ XT Index Kit (Illumina, San Diego, CA, USA). The library pool was sequenced on Illumina MiSeq platform (2 × 250 paired-end) using MiSeq Reagent Kit v2 (Illumina, San Diego, CA, USA) with 500-cycle paired-end reads.

The 16S rRNA transcript levels in the isolated DNA probes were determined by the 2^−ΔΔCT^ method [34] using the real-time PCR kit with SYBR Green I dye with manufacturer’s recommendations (Syntol, Moscow, Russia), where 1 was the amplification level without adding endophytes. Primers designed for qPCRs are shown in the Supplementary Table S4.

### 4.4. Bioinformatics Data Analysis

The bioinformatic analysis utilized data available in Tables S1–S3, with processing carried out using custom R scripts (https://github.com/niknit96/Aleynova_et.al.2025_2, accessed 6 October 2025). For 16S data, initial reads were preprocessed via QIIME 2 (version 2024.10.1) [35] and DADA2 (version 2024.10.0) [36] using the denoise-paired command with parameters -p-trunc-len-f 226 and -p-trunc-len-r 215, including primer removal with the QIIME 2 cutadapt plugin (version 2024.10.0) [37], chimera filtering, and merging of paired-end reads in DADA2. In the case of ITS data, primers were removed using cutadapt, and only forward reads were used in the DADA2 denoise-single step (with -p-trunc-len-f 195) due to poor reverse-read quality (Phred quality scores < 20). Taxonomic identification was conducted using the QIIME 2 BLAST+ algorithm (version 2024.10.0), leveraging the SILVA 138 database for 16S sequences [38] and the UNITE database for ITS sequences [39].

For initial filtering and data preparation, we employed several R packages—phyloseq (version 1.50.0) [40], microeco (version 1.16.0) [41], microViz (version 0.12.7) [42], and tidyverse (version 2.0.0) [43]. We excluded sequences corresponding to mitochondria, chloroplasts, non-bacteria, and non-fungi from the dataset. To account for uneven sequencing depth, samples were rarefied to a uniform depth of 5000 reads per sample. For alpha-diversity analysis (Shannon index), calculations were performed on the rarefied data without additional ASV filtering to preserve the representation of rare taxa. In contrast, for beta-diversity analysis (Bray–Curtis dissimilarity), a relative abundance threshold of >0.01% was applied to filter ASVs, thereby reducing potential noise from transient or artefactual sequences, focusing the analysis on the core community. To compare alpha diversity across treatments, we applied an analysis of variance (ANOVA). Principal coordinates analysis (PCoA) was performed on Bray–Curtis dissimilarity data. Beta diversity data were statistically validated using a permutation-based analysis of variance (PERMANOVA) with 999 permutations. All diversity metrics and associated statistical analyses were computed using the microeco R package [40] for both bacterial and fungal communities.

Visualization of results was carried out with microeco, microViz, tidyverse and Microsoft Excel; specifically, microeco and tidyverse were used to generate Figure 2, Figure 3 and Figure 6a,b, microViz was utilized for Figure 4 and Figure 7, and Microsoft Excel was used for Figure 1 and Figure 5.

## Data Availability

Raw sequences of the 16S rRNA and ITS1 rDNA regions have been deposited in the NCBI BioProject database under accession number PRJNA1437872. The data presented in this study are available within the article and Supplementary Materials.

## Supplementary Materials

The following supporting information can be downloaded at: https://www.mdpi.com/article/10.3390/plants15081151/s1, Table S1: 16S and ITS data samples used in analysis. Table S2: Classification bacterial ASV using consensus-blast. Table S3: Classification fungal ASV using consensus-blast. Table S4: Primers used in real-time PCR. Figure S1. Design of primers used for metagenomic sequencing. Figure S2. Amplification of the 16S rRNA gene in the DNA extracted from plants, treated by endophytes.
